# Concentration-Dependent Antibacterial Activity of Chitosan on *Lactobacillus plantarum*

**DOI:** 10.3390/pharmaceutics15010018

**Published:** 2022-12-21

**Authors:** Renátó Kovács, Lóránd Erdélyi, Ferenc Fenyvesi, Noémi Balla, Fruzsina Kovács, György Vámosi, Ágnes Klusóczki, Alexandra Gyöngyösi, Ildikó Bácskay, Miklós Vecsernyés, Judit Váradi

**Affiliations:** 1 Department of Medical Microbiology, Faculty of Medicine, University of Debrecen, 4032 Debrecen, Hungary; 2 Faculty of Pharmacy, University of Debrecen, 4032 Debrecen, Hungary; 3 Department of Pharmaceutical Technology, Faculty of Pharmacy, University of Debrecen, Nagyerdei Körút 98, 4032 Debrecen, Hungary; 4 Doctoral School of Pharmaceutical Sciences, University of Debrecen, 4032 Debrecen, Hungary; 5 Department of Pharmacy, Petz Aladár University Teaching Hospital, 9024 Győr, Hungary; 6 Department of Biophysics and Cell Biology, Faculty of Medicine, University of Debrecen, 4032 Debrecen, Hungary; 7 Institute of Healthcare Industry, University of Debrecen, 4032 Debrecen, Hungary; 8 Department of Pharmacology, Faculty of Pharmacy, University of Debrecen, 4032 Debrecen, Hungary

**Keywords:** chitosan, *Lactobacillus*, antibacterial, interaction, time-kill

## Abstract

The antimicrobial effect of chitosan and synthetic chitosan derivatives has been confirmed on many Gram-positive and Gram-negative bacteria and fungi. The tests were carried out on pathogenic microorganisms, so the mechanism and concentration dependence of the inhibitory effect of chitosan were revealed. We conducted our tests on a probiotic strain, *Lactobacillus plantarum*. Commercially available chitosan derivatives of different molecular weights were added to *L. plantarum* suspension in increasing concentrations. The minimum inhibitory concentration (MIC) value of chitosan was determined and confirmed the viability decreasing effect at concentrations above the MIC with a time-kill assay. The release of bacterium cell content was measured at 260 nm after treatment with 0.001–0.1% concentration chitosan solution. An increase in the permeability of the cell membrane was observed only with the 0.1% treatment. The interaction was also investigated by zeta potential measurement, and the irreversible interaction and concentration dependence were established in all concentrations. The interaction of fluorescein isothiocyanate (FITC) labeled low molecular weight chitosan and bacterial cells labeled with membrane dye (FM^®^ 4–64) was confirmed by confocal microscopy. In conclusion, the inhibitory effect of chitosan was verified on a probiotic strain, which is an undesirable effect in probiotic preparations containing chitosan additives, while the inhibitory effect experienced with pathogenic strains is beneficial.

## 1. Introduction

Chitosan is the precursor of chitin, which is the second most common polymer in nature after cellulose. It is presented in large quantities in the exoskeleton of insects or in the shell of crustaceans. Chitin is a polymer formed by random β- (1–4) glycosidic bonds of D-glucosamine and N-acetyl-glucosamine monomers whose deacetylation derivative is chitosan [1,2].

The ratio of the monomers affects the average molecular weight, determines the physico-chemical properties of the polymer, thus influencing its solubility in water and biological activity. The parameter generally specified in the chitosan specification is the degree of deacetylation, which is given by the manufacturers as a percentage. The degree of deacetylation shows the proportion of free amino groups in the molecule, which in the case of commercial chitosan is usually 40–75% [2,3]. The greater the degree of deacetylation, the better the water solubility of chitosan and the stronger the biological effect associated with it. In addition, the increase in the concentration of free amino groups and the increased protonation of amino groups in the acidic medium also increase the water solubility and biological efficiency of chitosan [2,3].

Chitosan is widespread as an auxiliary material in pharmaceutical technology, it is an excellent gel-forming and coating agent [4,5]. However, chitosan also has biological activity. Its antimicrobial effect on fungi and Gram-negative and Gram-positive bacteria has been confirmed [4,6]. It was proved that the polycationic structure is a condition for the antibiotic effect, since the antibiotic effect decreases with the reduction in the number of positive charges and the number of monomeric units. As the polymer chain shortens, the molecules lose their conformation and thus their bioactivity. However, as the chitosan chain becomes longer, the molecule rolls up, which reduces the possibility of interaction with the cell membrane [7].

The antimicrobial effect of chitosan on Gram-negative bacteria was investigated thoroughly, and its mechanism of action was confirmed in the last decade [8,9]. Because of its size, high molecular weight chitosan cannot enter the cell, so it mainly interacts with the cell extracellularly, e.g., blocking or rupturing the cell membrane as a polycation [8]. Lower molecular weight chitosan can enter cells and interact predominantly intracellularly [10]. Two types of mechanisms have been confirmed; depending on the conformation of chitosan, it can enter the cell by forming pores on the cell membrane or by endocytosis [11,12]. The chitosan enters the cytosol, binds to the proteins, and changes their conformation. The cell membrane forms an important barrier during exposure to chitosan. If the integrity of the membrane is damaged, substances in the cytosol are released, and these intracellular components can be easily detected by light absorption at 260 nm (nucleic acids) and 280 nm (proteins) [11,12,13]. Transmission electron microscopy (TEM) studies showed that pore formation on the bacterial cell surface indicated that 50 kDa 0.1% chitosan had lytic rather than static activity against *Escherichia coli*. The TEM photos confirmed that chitosan damaged the cell membrane of the cells [14].

The most often used Gram-positive bacteria test strain in the investigation of synthetic chitosan derivatives is *Staphylococcus aureus* [15,16]. It is often responsible for the development of wound infections, so in the case of many gel formulations, chitosan-based preparations are tested on it [17,18,19].

The mechanism of antibacterial activity of chitosan has not been examined on probiotic bacteria in detail yet. Only the minimum inhibitory concentration (MIC) values were determined for 28–1670 kDa molecular weight chitosan assayed by H.K. No et al. MIC values were between 0.1–0.05% for *Lactobacillus plantarum* [20]. 

The aim of our study is to investigate the concentration dependence and mechanism of chitosan’s antimicrobial effect on a probiotic, Gram-positive bacterium, *Lactobacillus plantarum*. In our previous studies, we found that during the dissolution test of probiotic microcapsules containing *L. plantarum* and coated with chitosan, a chitosan concentration close to the reported MIC concentration was dissolved in the release medium [21]. Thus, we first wanted to determine the MIC value of chitosan on *L. plantarum* for different molecular weights and to investigate the concentration dependence of the inhibition in the time-kill assay. The surface interaction was examined with a confocal microscope, and was evaluated based on the membrane permeability test and the zeta potential values.

## 2. Materials and Methods

### 2.1. Materials

*Lactobacillus plantarum* subsp. *plantarum* (ATCC 14917) was ordered from ATCC (Manassas, VA, USA). Low molecular weight chitosan (50–190 kDa; 20–300 cP; LMW), medium molecular weight chitosan (200–800 cP; MMW), high molecular weight chitosan (310–375 kDa; 800–2000 cP; HMW), phosphate-buffered saline (PBS), Hanks’ Balanced Salt Solution (HBSS), and formaldehyde 37% solution were purchased from Sigma-Aldrich (Budapest, Hungary). SYTOX Green Nucleic Acid Stain for flow cytometry and FM^®^ 4–64 dye were purchased from ThermoFischer Scientific (Budapest, Hungary). FITC-LMW chitosan (LMW*)was synthesized based on our previous publication [21].

For all investigations, chitosans with different molecular weights were dissolved in 10 *w*/*w*% acetic acid, and 1.0 *w*/*v*% chitosan solutions were prepared. Then the dilutions were performed with a sterile 0.9 *w*/*w*% sodium chloride solution and the pH was set to 6.5.

### 2.2. Determination of MIC Values

The susceptibility of *L. plantarum* to low molecular weight chitosan, middle molecular weight chitosan, and high molecular weight chitosan was determined in the brain heart infusion medium (BHI) (CliniChem Ltd., Budapest, Hungary). The concentrations tested ranged from 0.0004 to 0.25% (*v*/*v*) for all types of chitosan. Fresh *L. plantarum* culture grown overnight in the BHI medium was used to prepare the bacterial suspension in 0.9% NaCl (final optical density at 600 nm was 0.5). Afterward, 100 μL of *Lactobacillus* suspension previously diluted 1:500 in BHI medium with 100 μL chitosan solution was mixed on a microplate (TPP, Transdingen, Germany). Then, plates were incubated for 48 h at 37 °C in the presence of 5% CO_2_. The MICs were read visually as the lowest concentration that exerts at least 50% growth inhibition compared with the untreated growth control and are presented as the median value of three independent experiments per isolate.

### 2.3. Investigation of Killing Kinetics by CFU Determination

Based on the microdilution results, the killing activity of various chitosan compounds was determined in BHI at chitosan concentrations of 0.125%, 0.015%, and 0.003% in a final volume of 10 mL BHI. The starting inocula were 4–4.5 × 10^4^ CFU/mL. Aliquots of 100 μL were removed at 0, 4, 8, 12, and 24 h; afterwards, samples were serially diluted ten-fold and plated (4 × 30 μL) onto an MRS (Man, Rogosa and Sharp) medium (Merck, Budapest, Hungary) and incubated at 37 °C for 48 h in 5% CO_2_. Different chitosan concentrations were considered bactericidal when a ≥3log_10_ decrease in living bacterial cell number was caused, compared with the initial inocula.

### 2.4. Investigation of Live/Dead Cell Ratio and Cell Number by Flow Cytometry

All samples obtained by the time-kill assay (Section 2.3) were also analyzed by flow cytometry. Samples were centrifuged at 1000 rpm for 1 min, the supernatants were collected, and the bacterial cells were stained with SYTOX green reagent at 1 µM final concentration for 30 min at 37 °C. Samples were analyzed with a Guava Easy Cyte 6HT-2L flow cytometer (Merck Ltd., Darmstadt, Germany). Using green (525/30 nm) and red (695/50 nm) fluorescence channels, cells were gated out on a green versus red dot plot. Live/dead bacterial cell ratios were evaluated.

### 2.5. Membrane Integrity Assay

The membrane integrity of *L. plantarum* was studied by measuring the released materials at 260 nm as published previously [13]. Bacterium pre-culture was grown in 5 mL BHI medium at 37 °C for 18 h at 2.3 Hz shaking frequency, diluted to the optical density of 0.6 at 640 nm (OD640) with 0.9% NaCl sterile solution. Different concentrations of 0.1%, 0.01%, and 0.001% (*w*/*v*) of LMW, MMW and HMW chitosan solutions were added to the bacterium suspension to the ratio of 1:1 (*v*/*v*). The release of metabolic substances was investigated with Multiskan Go (ThermoFisher) microplate reader detection at 260 nm every 10 min for 180 min.

### 2.6. Investigation of Chitosan Interaction by Confocal Laser Scanning Microscopy

Different concentrations of FITC-chitosan solutions (0.1%, 0.01%, 0.001% (*w*/*v*)) were mixed with bacterium suspension at the ratio of 1:1 (*v*/*v*) and samples were incubated at 37 °C for 60 min. Then samples were washed twice with sterile PBS to remove the excess FITC-chitosan and centrifuged (11,000× *g*, 10 min, 25 °C). The bacterium cell membrane was stained with 5 μg/mL ice-cold FM^®^ 4–64 dye and then fixed with ice-cold 4% formaldehyde in HBSS on ice for 10 min. The bacterium suspension OD was adjusted as the same described in Section 2.5.

Confocal images were recorded with a Zeiss LSM 880 confocal microscope (Carl Zeiss, Jena, Germany) equipped with a Plan-Apochromat 63x NA 1.4 oil immersion objective. FITC was excited at 488 nm with an Ar-ion laser (set to 1%) and detected between 499–544 nm, whereas FM^®^ 4–64 was excited with a 543-nm HeNe laser (30%) and detected in the range of 600–700 nm. Image parameters were the following: 256 × 256 pixels, pixel size 53 nm, zoom 10×, and pinhole set to 1 Airy unit resulting in an optical slice thickness of 0.7 μm in the green and 0.8 μm in the red channel; z-step size during sectioning was 0.37 μm, pixel dwell time was 4.1 or 16.5 μs, and each line of the image was scanned 4× and averaged. Crosstalk between the channels was excluded by sequentially exciting the dyes and switching line by line. From each z-stack, one slice was selected for display.

### 2.7. Determination of Zeta Potential on Bacterial Cell Surface

*L. plantarum* suspensions were prepared according to the method of membrane integrity assay (2.4.). Diluted LMW, MMW, and HMW chitosan solutions were mixed in concentrations of 0.1%, 0.01%, and 0.001% with *L. plantarum* suspension to the ratio of 1:1 (*v*/*v*) and incubated at 37 °C for 60 min. Samples were washed as described in Section 2.6. method to remove the excess chitosan from the suspension. Zeta potentials were measured in high concentration zeta potential cells by Malvern Nano-ZS Zetasizer (Malvern Instruments, Malvern, UK). Data were collected and analyzed with GraphPad Prism 5.0 software.

### 2.8. Statistical Analysis

For statistical analyses, GraphPad Prism 5.0 software (GraphPad Software Inc., La Jolla, CA, USA) was used. Data are presented as means ± SD. Comparisons of groups were performed using one-way and two-way ANOVA and Bonferroni multiple comparison tests. Differences were considered not significant at *p* > 0.05; significant at *** *p* < 0.001 and ** *p* < 0.01.

## 3. Results

### 3.1. MIC Values and Time-Kill Assay

**Figure 1:**
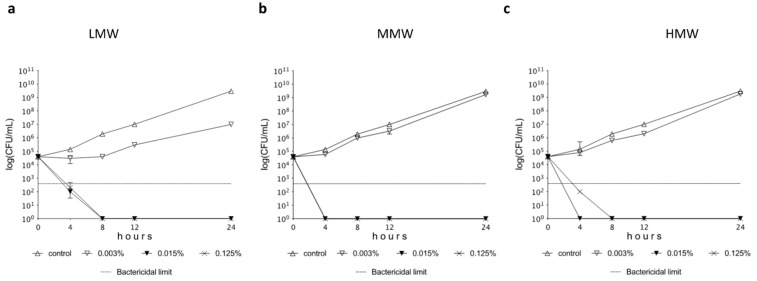
Time-kill plots of *L. plantarum* following exposure to 0.003–0.125% LMW (**a**), MMW (**b**), and HMW (**c**) chitosan solution in BHI medium. The investigation was done in triplicates.

MIC values of the tested chitosan compounds proved to be 0.007% in case of both LMW, MMW, and HMW variants. The killing kinetics of various types of chitosan are presented in Figure 1. All tested chitosan with different molecular weights exerted a marked inhibitory effect against *L. plantarum* in a concentration-dependent manner. A remarkable bactericidal effect (at least a three log decrease in living cell number compared to starting inoculum) was observed from 0.015% chitosan in the case of all molecular weights. 

### 3.2. Investigation of Live/Dead Cell Ratio by Flow Cytometry 

**Figure 2:**
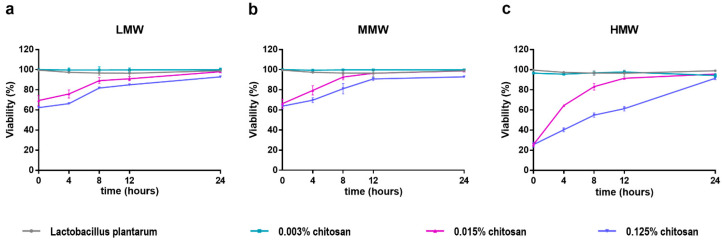
Viability of *L. plantarum* after treatment with different concentration of low molecular weight (LMW) chitosan (**a**), medium molecular weight (MMW) chitosan (**b**), and high molecular weight (HMW) chitosan (**c**). The investigation was done in triplicates.

The viability of the bacterial cells, based on the ratio of live/dead cells, was determined with a flow cytometer (Figure 2). No significant difference in the viability of *L. plantarum* was observed in the case of the control and the samples with the lowest (0.001%) chitosan concentration. This can be established in the examination of all three molecular weights of chitosan. However, with a tenfold increase in chitosan concentration (0.01% and 0.1%), a significant decrease in viability was detected. Regarding the molecular weight of chitosan, the viability showed a decreasing trend as the molecular weight increased. The live/dead cell ratio stabilized at 8 h for LMW, but only at 12 h for MMW. In the case of HMW, stabilization was observed in the 24 h result.

### 3.3. Membrane Integrity Assay

**Figure 3:**
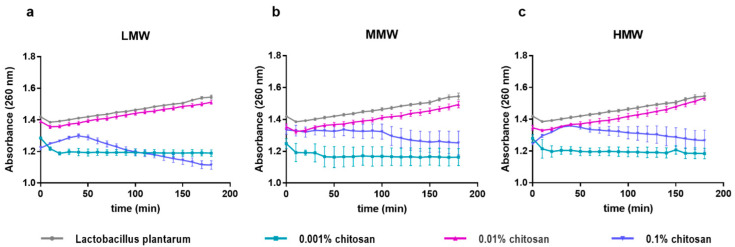
The release of cell content of *L. plantarum* was measured at 260 nm after treating with 0.001–0.1% concentration of LMW chitosan (**a**), MMW chitosan (**b**), and HMW chitosan (**c**).

The destabilizing effect of the chitosan on the membrane was investigated in the integrity test of the *L. plantarum* membrane, and the amount of released nucleic acid was measured at 260 nm. In the case of untreated bacterium cells, we experienced a small, linear increase in absorbance, which can be seen in Figure 3. However, by increasing the concentration of chitosan, we found a significant difference between some curves. We detected a lower absorbance at a concentration 0.001% compared to the control for all three chitosan molecular weights. During the treatment at a concentration of 0.1%, we found a maximum in the absorbance of the samples at 30–40 min, then we measured a decreasing trend. These characteristics are more pronounced in the case of low and high molecular weight samples, while less so in the case of medium ones. The absorbance of the samples at a concentration of 0.01% changed similarly to the untreated control. 

### 3.4. Determination of Zeta Potential on Bacterial Cell Surface 

**Figure 4:**
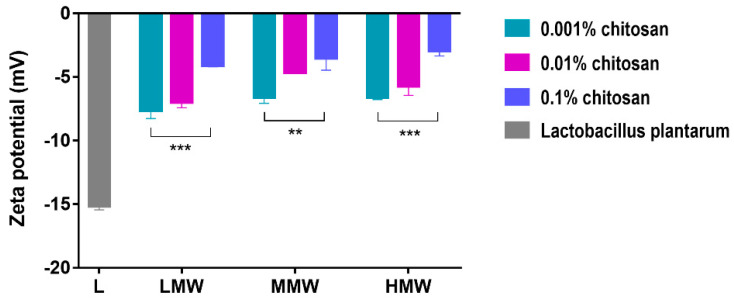
Zeta potential values of untreated (L) *Lactobacillus plantarum* and 0.001–0.1% LMW, MMW, and HMW chitosan treated bacterium cell surface. Zeta potential showed negative values. The investigation was done in triplicates; data are presented as means ± SD; *** *p* < 0.001 and ** *p* < 0.01.

The zeta potential was determined on the surface of *L. plantarum*, results shown in Figure 4. The value of the zeta potential was −15.15 ± 0.49 mV in untreated *L. plantarum* containing samples, which became more positive after the chitosan treatment. The zeta potential showed concentration dependence for all three molecular weight chitosan. The largest zeta potential change was obtained with the 0.1% chitosan treatment (LMW: −4.10 mV, MMW: −3.52 mV, HMW: −2.96 mV). Comparing the results obtained with chitosan of different molecular weights, no significant difference was observed in the zeta potential values. Statistical analysis was performed with two-way ANOVA and Bonferroni multiple comparison tests.

### 3.5. Investigation of Chitosan Interaction by Confocal Laser Scanning Microscopy

**Figure 5:**
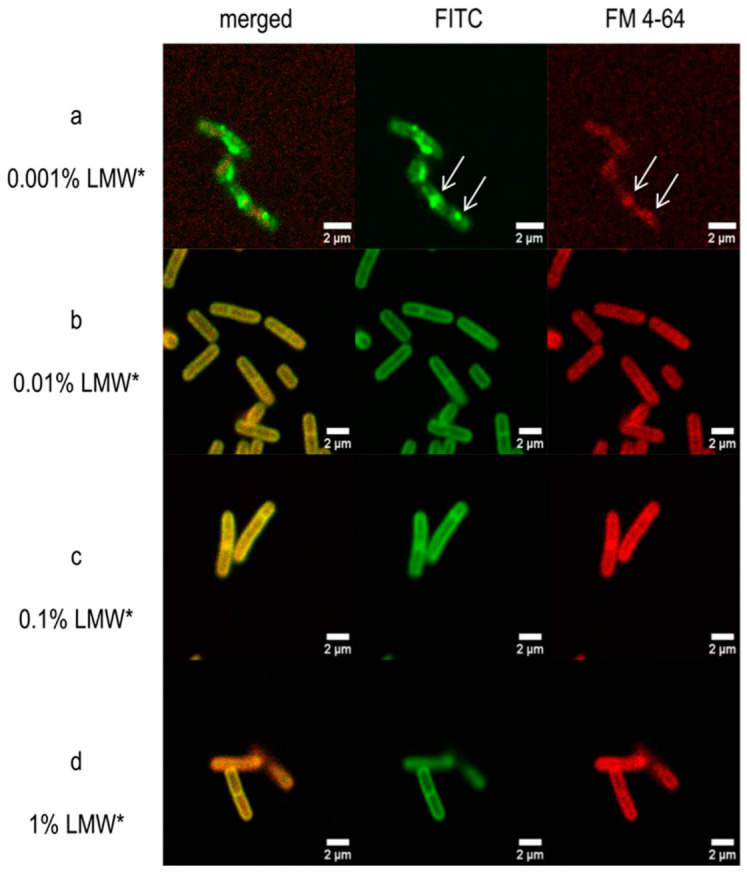
Confocal microscopic images of *L. plantarum* after treatment with 0.001% (**a**), 0.01% (**b**), 0.1% (**c**), and 1% FITC labeled LMW chitosan (LMW*) (**d**). On the second column, the LMW* is shown in green, on the third column the FM^®^ 4–64 dye is shown in red, and on the first column merged channels are demonstrated. White arrows show membrane aggregations.

*Lactobacillus* cells were treated with chitosan concentrations used in other experiments (0.001–0.1%), and treatment with a higher concentration (1%) was also used. In the case of the lowest chitosan concentration, an inhomogeneous distribution of LMW* was observed in the bacterial cells, in the proximity of the cell wall, while the FM^®^ 4–64 used for membrane staining also showed an uneven distribution, which suggests membrane aggregation (on Figure 5 shown with arrows). At the lowest concentration, not only was membrane aggregation observed, but also the altered shape of the cells indicates cellular damage. Chitosan is evenly distributed on the surface of the bacterial cell wall at higher concentrations (0.01–1%). In addition to uniform staining of the membrane, normal morphology of cells without any membrane damage was observed.

## 4. Discussion

In our previous study, chitosan was used as a coating agent during the development of probiotic microcapsules, and we experienced a significant decrease in viability in the dissolution tests [21]. The concentration of dissolved chitosan was determined in the dissolution medium, which was compared with the MIC values reported in the literature [20]. We found that the 0.043% concentration of chitosan in the dissolution fluid corresponds with the literature value, so MIC values of the commercially available chitosan used as a coating agent and gel-forming material were determined. In this study, chitosan with three different molecular weights was used: low molecular weight chitosan (50–190 kDa; 20–300 cP; LMW), medium molecular weight chitosan (200–800 cP; MMW), and high molecular weight chitosan (310–375 kDa; 800–2000 cP; HMW). In this study, 0.007% of MIC values were determined for all chitosan products with different molecular weights. Thus, our previous assumption that chitosan dissolves in the release medium at a concentration above the MIC value was confirmed.

We also wanted to prove our hypothesis with further tests, as well as investigate the nature of the antibacterial effect experienced in the case of *L. plantarum*. Time-kill curves (Figure 1) monitor the effect of different concentrations of various molecular weight chitosan over time in relation to stages of growth of *L*. *plantarum*. A bactericidal effect was observed above the MIC concentration of 0.007% for all three molecular weight chitosans. We did not experience a bactericidal effect at the lowest concentration (0.015–0.003%), which correlates with the MIC value. The samples inoculated in the time-kill experiment were also examined with a flow cytometer, and the viability was determined by fluorescent labeling of dead *Lactobacilli* (Figure 2). Our results here are also consistent with the time-kill assay. The viability of *L. plantarum* was not reduced in the case of treatment with the lowest concentration (0.003%), but at higher concentrations (0.015–0.125%) it was reduced by chitosan to 25–75%. A time-kill test was not performed for chitosan in the case of *Lactobacillus* strains, but a gallic acid–chitosan derivative was tested on *S. aureus* and *E. coli* [22]. On these time-killing curves, the bacteria were treated with multiples of the MIC value, which clearly shows that the concentration above the MIC resulted in the death of the bacteria.

The interaction of the bacterial cell wall with certain substances can lead to the release of cytoplasmic substances. These interactions reduce the integrity of the membrane, during which potassium and phosphate ions, which are found at higher concentrations in the cytoplasm, are first released, and then larger molecules are also released. The effect of chitosan on membrane permeability has also been investigated in different Gram-negative bacteria (*P. aeruginosa* and *E. coli*) [14]. In the case of *E. coli*, the release of nucleic acids and proteins at 260 and 280 nm was followed as a function of time. When the nucleic acid is released after 30 min, the time curve has a maximum after 40 min under the influence of 0.1% 50 kDa and 5000 kDa chitosan and then shows a decreasing trend after 100 min. Liu et al. investigated the damage of cell membranes by chitosan to *S. aureus*, which is a Gram-positive pathogen bacterium, and found that the process is concentration-dependent. They certified it with TEM micrographs, and where *S. aureus* was treated with 0.5% chitosan, the membrane of dividing cells was disrupted with the loss of cell contents [23]. Our results show a similar tendency, having a maximum on the curves after mixing the *L. plantarum* cell suspension and 0.1% chitosan solutions (Figure 3). We performed tests also with 0.01% and 0.001% chitosan solutions, in which case we experienced a different tendency. A low absorbance was measured for the addition of the 0.001% chitosan solution, and in the medium concentration (0.01%) absorbance similar to the control was measured, but there was no maximum peak on these curves. This indicates that the cell wall of the bacterium becomes permeable only for the 0.1% treatment, which, based on the above time-kill results, is probably due to a bactericidal effect. In the lower concentration treatments, we did not experience cytoplasmic nucleic acid release, which indicates a bacteriostatic interaction in the 0.01% treatments.

In this study, we did not observe a significant difference in nucleic acid release between chitosan products with different molecular weights; however, the maximum prolonged on the MMW chitosan curve, the nucleic acid release is lower compared to LMW and HMW. This is probably because MMW chitosan is a physical mixture of LMW and HMW, which gives a medium viscosity product. In the case of MMW chitosan, we generally experienced a larger deviation in the results, which is presumably also due to the above reason.

Based on the permeability test, we assumed that a concentration-dependent interaction occurs between the Gram-positive cell wall of *L. plantarum* and chitosan. So far, permeability studies have been performed on Gram-negative pathogen bacteria, so we aimed to get information on the mechanism of interaction and inhibition more thoroughly in the case of *L. plantarum*, as a Gram-positive bacterium. Due to the polycationic nature of chitosan, it is expected that the negatively charged bacteria will interact with chitosan [24]. The main component of the Gram-positive bacteria cell wall is the peptidoglycans, which are covalently linked wall teichoic acids (WTAs) and lipoteichoic acids (LTAs). These are anionic polymers, WTAs, in Gram-positive bacteria that can make up almost half of the total dry weight of the cell wall and, thus, can provide high-density negative charges in the cell wall [25,26].

By measuring the zeta potential, we examined the extent to which this interaction affects the surface of the bacterial cell wall. In the experiments, the bacterial suspension and the chitosan solutions were mixed. After the incubation, the liquid medium was removed from the samples by centrifugation, the cell suspension was washed with physiological salt solution, and then the zeta potential was measured. The zeta potential results can establish that the chitosan remained on the surface even after the washing of samples, because the surface of the bacterial cells became more and more positive as the concentration of chitosan increased (Figure 4). This suggests that the higher the concentration of chitosan, the more it covers the surface of the bacteria. At higher concentrations, due to the entanglement of the polymer chains, chitosan here behaves as if it were a long chain with a high molecular weight. Due to its large size, it cannot enter the cell just interacts extracellularly. Probably this long-lasting interaction allows the bacterial cell membrane to open and increase its permeability [11,27]. 

The interaction was examined using a confocal microscope with fluorescent labeling of the membrane and FITC-LMW chitosan. FM^®^ 4–64 was used as a membrane dye, which is also used to label endosomes [28]. At a lower concentration (0.001%), chitosan is not evenly distributed on the surface of the bacterial cell, which is indicated by the green patches of FITC-chitosan (Figure 5). In addition, similar aggregates were observed in the bacterial membrane (red color), which overlap with the FITC-chitosan aggregates. The thickened membrane structure in places may also indicate internalization, occurring at low concentrations, as a result of chitosan entering the bacterial cell [11,27]. However, at 0.001% concentration, it probably does not result in a bactericidal or bacteriostatic effect either since we did not observe a significant decrease in CFU values at the concentration lower than MIC. Nevertheless, in the low molecular weight chitosan samples, the CFU is slightly low at the beginning of the assay, which may indicate some inhibitory effect.

## 5. Conclusions

Based on our previous experience, we investigated the background of the viability decreasing effect of chitosan on *L. plantarum*. Research shows that it has an inhibitory effect on the division of bacterial cells in the case of Gram-negative and Gram-positive bacteria, and, depending on the concentration, it ruptures the cell membrane and releases substances from the cytosol. The antimicrobial activity of chitosan is realized by different mechanisms depending on the concentration and molecular weight of chitosan. Studies related to the antimicrobial effect of chitosan have so far understandably covered pathogenic microorganisms, which raises the possibility of its therapeutic application. Many chitosan derivatives are synthesized with the aim of increasing the solubility of chitosan, thereby increasing its antimicrobial properties. This is an important and useful task in the fight against antibiotic resistance. However, with our studies, we proved that knowledge of this interaction is important not only for pathogenic microorganisms but also for probiotic flora. Therefore, in the case of the development of probiotic preparations, we recommend determining the MIC value of the excipients used. In view of the inhibitory concentration, we can draw relevant conclusions about the dissolution test results as well as the effectiveness and stability of the preparation.

## Data Availability

Not applicable.
